# Singapore Grouper Iridovirus Disturbed Glycerophospholipids Homeostasis: Cytosolic Phospholipase A2 Was Essential for Virus Replication

**DOI:** 10.3390/ijms222212597

**Published:** 2021-11-22

**Authors:** Na Ni, Jiaying Zheng, Wenji Wang, Linyong Zhi, Qiwei Qin, Youhua Huang, Xiaohong Huang

**Affiliations:** 1 University Joint Laboratory of Guangdong Province, Hong Kong and Macao Region on Marine Bioresource Conservation and Exploitation, College of Marine Sciences, South China Agricultural University, Guangzhou 510642, China; 20193140010@stu.scau.edu.cn (N.N.); zjy19931203@stu.scau.edu.cn (J.Z.); wangwenji653750@stu.scau.edu.cn (W.W.); zly123@stu.scau.edu.cn (L.Z.); qinqw@scau.edu.cn (Q.Q.); 2 Guangdong Laboratory for Lingnan Modern Agriculture, Guangzhou 510642, China; 3 Southern Marine Science and Engineering Guangdong Laboratory, Zhuhai 519082, China

**Keywords:** cytosolic phospholipase A2, SGIV, glycerophospholipids, cyclooxygenase (COX), 5-Lipoxygenase (5-LOX)

## Abstract

Singapore grouper iridovirus (SGIV), belonging to genus *Ranavirus*, family *Iridoviridae*, causes great economic losses in the aquaculture industry. Previous studies demonstrated the lipid composition of intracellular unenveloped viruses, but the changes in host-cell glyceophospholipids components and the roles of key enzymes during SGIV infection still remain largely unknown. Here, the whole cell lipidomic profiling during SGIV infection was analyzed using UPLC-Q-TOF-MS/MS. The lipidomic data showed that glycerophospholipids (GPs), including phosphatidylcholine (PC), phosphatidylserine (PS), glycerophosphoinositols (PI) and fatty acids (FAs) were significantly elevated in SGIV-infected cells, indicating that SGIV infection disturbed GPs homeostasis, and then affected the metabolism of FAs, especially arachidonic acid (AA). The roles of key enzymes, such as cytosolic phospholipase A2 (cPLA2), 5-Lipoxygenase (5-LOX), and cyclooxygenase (COX) in SGIV infection were further investigated using the corresponding specific inhibitors. The inhibition of cPLA2 by AACOCF3 decreased SGIV replication, suggesting that cPLA2 might play important roles in the process of SGIV infection. Consistent with this result, the ectopic expression of EccPLA2α or knockdown significantly enhanced or suppressed viral replication in vitro, respectively. In addition, the inhibition of both 5-LOX and COX significantly suppressed SGIV replication, indicating that AA metabolism was essential for SGIV infection. Taken together, our results demonstrated for the first time that SGIV infection in vitro disturbed GPs homeostasis and cPLA2 exerted crucial roles in SGIV replication.

## 1. Introduction

Iridoviruses, members of nucleocytoplasmic large DNA viruses (NCLDV), not only cause great economic losses in the aquaculture industry but are emerging infectious disease agents showing a significant threat to global biodiversity [1,2]. Iridoviruses are capable of infecting invertebrates and vertebrates, such as insects, fish, amphibians, and reptiles [3]. To date, iridoviruses are divided into five genera: *Iridovirus*, *Chloriridovirus*, *Lymphocystivirus*, *Megalocytivirus*, and *Ranavirus* [3]. Similar to frog virus (FV) 3, a strain type of *Ranavirus,* most of the vertebrate iridovirus isolates shared the same overall replication strategy. Virus entry, assembly and release of iridovirus are intimately associated with the cell membrane system, which is composed of different lipid composition. The enveloped virions enter into cells by endocytosis, whereas non-enveloped virions enter by binding the plasm membrane with the subsequent release of the viral core into the cytoplasm [4,5,6]. As with other large DNA viruses, the interaction of the internal viral membrane with either the plasma or endosomal membrane was required for release of viral DNA cores [7]. During viral assembly, membrane-like structures served as scaffolds for the deposition of viral proteins [6,8]. Finally, mature enveloped virions were observed on the cell surface after budding from the plasma membrane of infected cells. Therefore, membrane remodeling exerted crucial roles during the life cycle of iridovirus infection.

An increasing number of studies demonstrated that membrane remodeling was manipulated by viruses at multiple stages of their life cycle, including lipid–receptor interactions, the fusion of viral envelopes with cellular membranes during endocytosis, the reorganization of cellular membranes to form replication compartments, and the envelopment and budding of virions [9]. The significant perturbations in the host-cell lipidomic profiles were demonstrated during the infection with different viruses, including dengue virus [10,11], human coronavirus 229E [12], rotavirus [13], Zika virus [14] and enterovirus [15]. The results from high-resolution mass spectrometry showed that the membrane remodeling occurring in dengue virus-infected cells was directly linked to a unique lipid repertoire [10]. As one of the major components of cellular membranes, glycerophospholipids (GPs), which are synthesized from glycerol-3-phosphate (G3P) in a de novo pathway, exert crucial roles in response to virus infection [16,17]. Moreover, key enzymes involved in GPs metabolism were reported to play an essential role in the process of membrane rearrangements during virus infection. For example, Phospholipase A2s (PLA2s), which catalyze the hydrolysis of GPs at the sn-2 ester bond to liberate lysophospholipid and arachidonic acid (AA), are involved in the replication and assembly of several RNA viruses [17,18,19,20,21,22]. During Hepatitis C virus (HCV), the knockdown of PLA2G4C not only suppressed viral replication and assembly, but also resulted in the formation of defective membranous web (MW) structure [20]. The cPLA2 also affected coronavirus RNA and protein accumulation via the production of lysophospholipids that were required for the formation of replicative organelles (ROs) [19]. Nevertheless, the role of PLA2 in fish virus replication was rarely reported.

Singapore grouper iridovirus (SGIV), a novel member of *Ranavirus*, was first isolated from diseased grouper (*Epinephelus tauvina*). This highly lethal and serious systemic disease induced by SGIV infection causes great economic losses in the grouper industry [23]. The results of electron microscopy showed that SGIV entry, assembly, and budding was closely associated with the plasma membranes [24]. During endocytosis, the SGIV envelope might be fused with endosome membrane for viral uncoating. In addition, as with other *Ranavirus*, the tubular membrane-like structures were observed in virus assembly sites in SGIV-infected cells [25]. Of note, the host-cell lipid dynamics in the context of SGIV infection were proposed as related to virus infection-induced apoptosis in GEC cells [26]. However, SGIV infection in several host cells, such as GS, GB, GK and GL evoked non-apoptotic cell death, and no apoptotic bodies were examined in infected cells [27,28]. Therefore, it is worthy to clarify the changes in host-cell lipid components in response to SGIV infection.

In the present study, the whole-cell lipidomic profiling during SGIV infection was analyzed using high-resolution mass spectrometry. The lipidomic results showed that glycerophospholipids (GPs) and fatty acids (FAs) were significantly elevated in SGIV-infected cells. Furthermore, the role of crucial enzymes involved in GPs or AA metabolism during SGIV infection were investigated using the corresponding specific inhibitors, including cPLA2, 5-Lipoxygenase (5-LOX), and cyclooxygenase (COX). These results extend the current understanding of the interaction between SGIV and host-cell glycerophospholipids, and shed important light on SGIV pathogenesis.

## 2. Results

### 2.1. Infection with SGIV Disturbed Glycerophospholipids (GPs) Homeostasis

**Figure 1:**
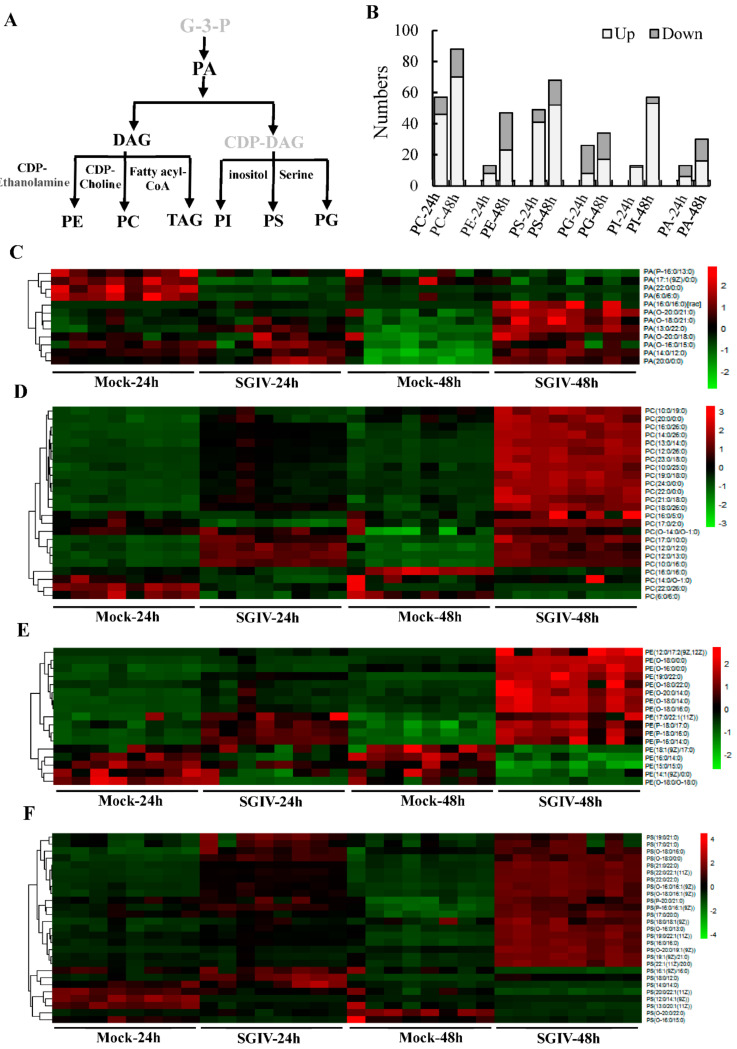
Lipidomic analysis of SGIV-infected cells showed a homeostatic change in glycerophospholipids (GPs). (**A**) The de novo biosynthetic pathways of GPs. (**B**) The number of lipid GPs subclasses detected in this study. (**C**–**F**) Heatmap of the differential changes of GP species, including phosphatidic acid (PA), phosphatidylcholine (PC), phosphatidylethanolamine (PE), phosphatidylserine (PS), which were all detected in SGIV-infected cells at 24 h and 48 h p.i. The heatmap generation was performed using the *R* package heatmap. Abbreviations: PG: glycerophosphoglycerol, PI: glycerophosphoinositols.

To gain insight into the changes of lipids components in SGIV replication, we performed an untargeted lipidomic analysis of GS cells upon SGIV infection using UPLC-Q-TOF-MS/MS. The abundance of 428 or 697 lipids were significantly changed in SGIV-infected cells at 24 h or 48 h, respectively. These differential lipids belonged to 32 subclasses within the five major class, including glycerophospholipids (GPs), glycerolipids (GLs), sphingolipids (SPs), Fatty Acyls (FAs) and Sterol Lipids (STs). Among those differential lipids, more than 40% of lipids belonged to GPs. Given that GPs are major components of cellular membranes, we paid special attention to the abundance of GPs within cells infected with SGIV, when compared to mock cells. Firstly, we showed the biosynthetic pathways of GPs via de novo synthesis, as described by Hishikawa et al. [29] (Figure 1A). Our lipidome analyses showed that the level of GP species began to change significantly at 24 h p.i., and the number of GP species increased with the infection time. A total of 176 and 332 species of GPs were significantly changed upon SGIV-infection at 24 h and 48 h, respectively. As shown in Figure 1B, the number of up-regulated or down-regulated GPs within different subclasses were counted. PC and PS, were the most varied subclass, followed by PI. The majority of differential species of PS, PC and PI were markedly upregulated (Figure 1D,F). For example, in 24 h or 48 h SGIV-infected cells, about 80.7% (46 of 57) or 79.55% (70 of 88) of PSs species increased in abundance (Figure 1F). In contrast, most of PG species decreased in abundance in SGIV-infected cells compared to those in mock cells. Additionally, the half of PE and PA species increased in abundance over infection time, and the other half of those species decreased (Figure 1C,E). Interestingly, there were no significant changes in the abundance of lyso-GPs during SGIV infection. The differently regulated GPs exhibited a wide variety of trends, suggesting that SGIV infection resulted in the alteration of GPs homeostasis.

### 2.2. Infection with SGIV Resulted in Alteration of Fatty Acyls (FAs)

**Figure 2:**
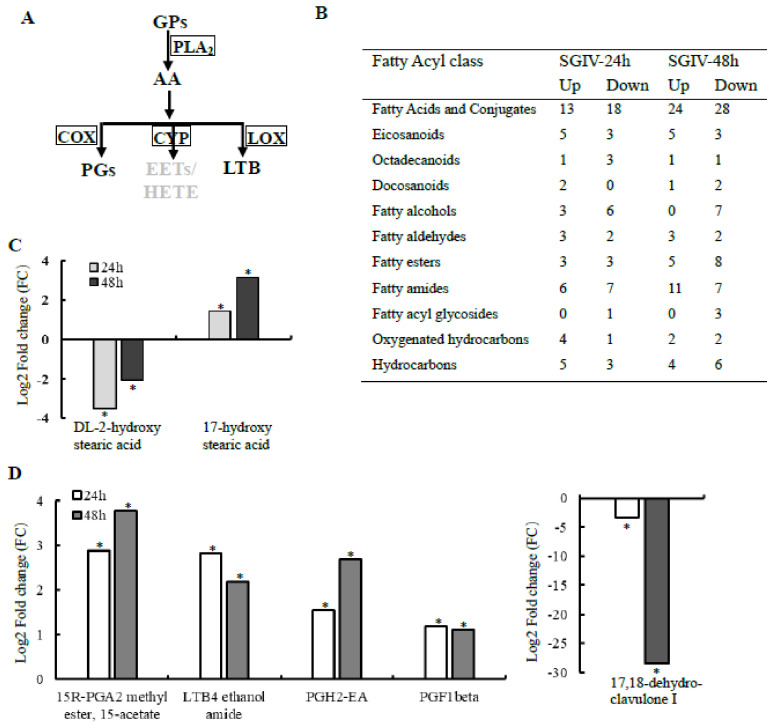
Lipidomic analysis showed that fatty acid (FAs) were significantly elevated in SGIV-infected cells. (**A**) The fatty acid remodeling of GPs. (**B**) The number of lipid species within 11 FAs subclasses that were significantly different between SGIV- and mock-infected cells. (**C**) The abundance of lipid molecules belonging to octadecanoids in SGIV-infected cells. (**D**) The abundance of lipid molecules belonging to eicosanoids in SGIV-infected cells. Asterisks (*) indicated a significant change in abundance between SGIV-infected and mock-infected cells. Abbreviations: PGA2: prostaglandin A2, PGH2: prostaglandin H2, PGF: prostaglandin F, LTB4: Leukotriene.

Glycerophospholipid acyl chains derived from de novo synthesis were subsequently remodeled by the cooperation of phospholipases (PLAs) and lysophospholipid acyltransferases (LPLATs) to generate lysophospholipids and lipid mediators, such as fatty acid derivatives [29]. Our lipidomic analyses also showed that SGIV infection resulted in alteration of fatty acids and derivatives. As shown in (Figure 2), 92 and 125 species of FAs were significantly changed upon SGIV infection at 24 h and 48 h, respectively. These changed FAs species belonged to 11 subclasses of FAs, including fatty acids and conjugates, eicosanoids, octadecanoids, docosanoids, fatty alcohols, fatty aldehydes, fatty esters, fatty amides, glycosides, oxygenated hydrocarbons, and hydrocarbons. Eicosanoids are inflammatory mediator molecules that have known functions in immunomodulation [30]. Among those differentially changed FAs, four species of FAs belong to eicosanoids, which have known functions in immunomodulation that were increased at both time points upon SGIV infection, including three prostaglandins (PGH2-EA, 15-keto-PGF2α and PGF1β), one leukotriene (LTB4 ethanol amide). While one thromboxane (2,3-Dinor-TXB1) showed a decreased level in SGIV-infected cells at 24 h, and one eicosanoid (17,18-dehydro-clavulone I) also showed a decrease in abundance at both time points upon SGIV infection. Two species of octadecanoids, 17-hydroxy stearic acid and DL-2-hydroxy stearic acid, showed different changes in abundance during SGIV infection. The level of the former increased, while the latter decreased (Figure 2C).

### 2.3. Pharmacological Inhibition of PLA2 Activity Affected SGIV Replication

**Figure 3:**
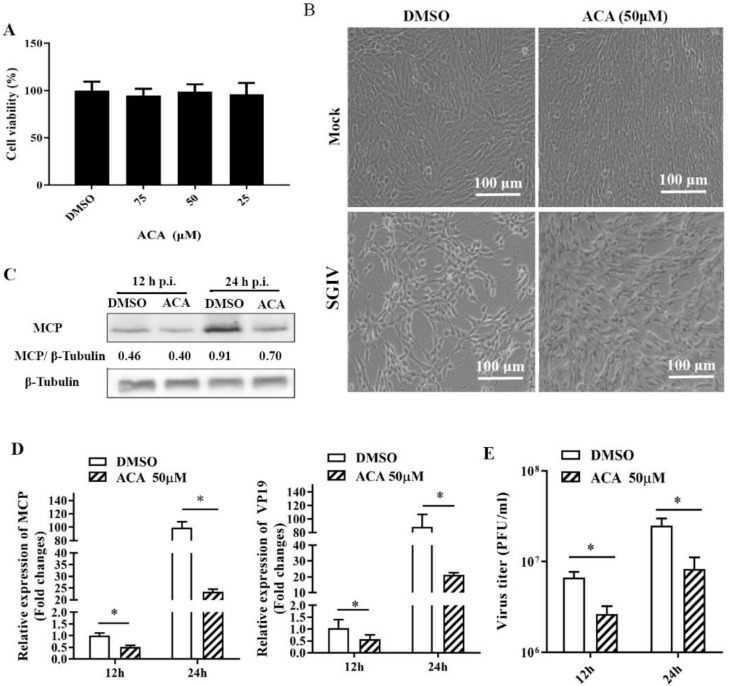
SGIV replication was inhibited by a broad-spectrum PLA2 inhibitor, Anthranilic acid (ACA), in vitro. (**A**) The cytotoxicity of ACA on GS cells was determined using MTT assay. (**B**) The severity of CPE induced by SGIV infection at 24 h p.i. in ACA-treated cells. (**C**) Western blotting analysis of viral major capsid protein (MCP) during SGIV infection in ACA-treated cells. (**D**) The effect of ACA on viral gene transcription during SGIV infection. (**E**) The effect of ACA on virus titers during SGIV infection. The asterisks (*) indicated *p* < 0.05.

It was reported that phospholipase A2 (PLA2) catalyzed the hydrolysis of the sn-2 position of membrane glycerophospholipids to liberate arachidonic acid (AA), which is a precursor of eicosanoids [18]. Given that the levels of glycerophospholipids and AA were altered by SGIV infection, we speculated that the activity of the host enzyme PLA2 might be involved in these alterations in lipid biosynthesis during SGIV infection. Using ACA (N-(P-amylcinnamoyl) anthranilic acid), a broad-spectrum inhibitor of PLA2 activity, we assessed the effects of ACA on virus replication. First, the non-cytotoxic concentration of ACA was evaluated, and the results showed that cell viability was not affected by ACA concentrations of up to 50 μM (Figure 3A). Next, the effects of ACA on SGIV infection were determined. As shown in Figure 3B, treatment with ACA obviously delayed the cytopathic effect (CPE) induced by SGIV at 24 h p.i. Significant reductions in viral gene transcription and viral protein synthesis were determined in the presence of ACA during SGIV infection (Figure 3C,D). Furthermore, treatment with ACA decreased the viral titers up to 40.03% or 33.2% at 12 h or 24 h, respectively (Figure 3E).

**Figure 4:**
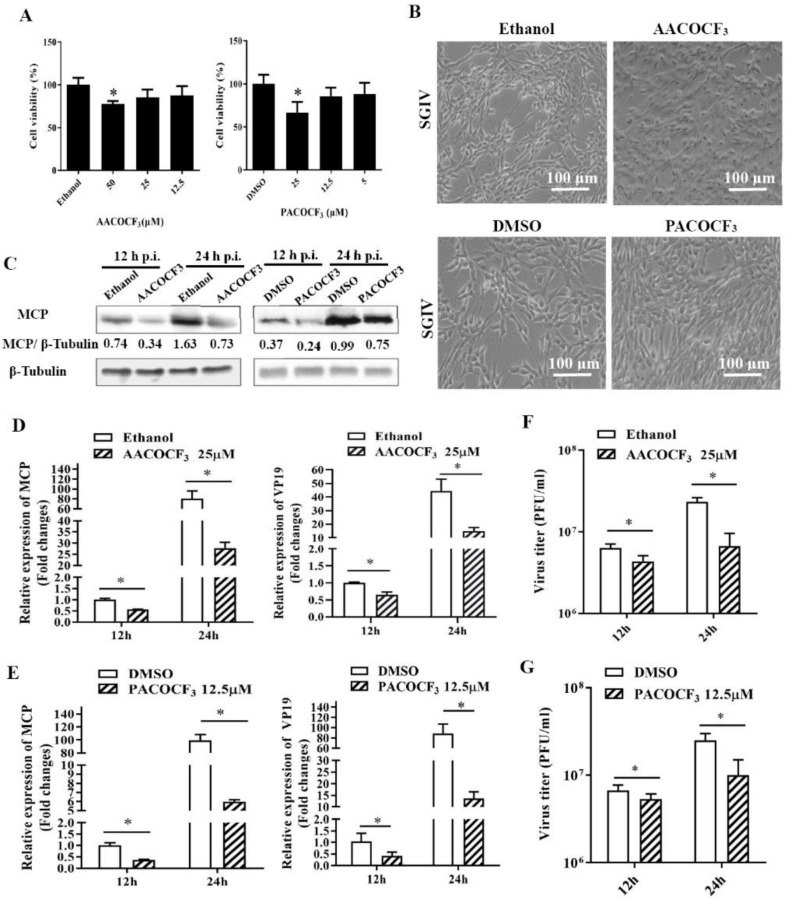
Production of infectious SGIV progeny in cell culture was inhibited by the cPLA_2_ inhibitor AACOCF3 or iPLA_2_ inhibitor PACOCF_3_. (**A**) The cytotoxicity of AACOCF3 or PACOCF_3_ on GS cells was determined using MTT assay. (**B**) The severity of CPE induced by SGIV infection at 24 h p.i. in AACOCF3-treated or PACOCF_3_-treated cells. (**C**) Western blotting analysis of viral major capsid protein (MCP) during SGIV infection in AACOCF3-treated or PACOCF_3_-treated cells. (**D**,**E**) The effects of AACOCF3 or PACOCF3 on viral gene transcription during SGIV infection. (**F**,**G**) The effects of AACOCF3 or PACOCF_3_ on virus titers during SGIV infection. The asterisks (*) indicated *p* < 0.05.

Given that PLA2 superfamily comprised several main types such as the cytosolic cPLA2 and calcium-independent iPLA2, as well as secreting sPLA2 [30], we selected specific inhibitors of iPLA2 (PACOCF_3_) and cPLA2 (AACOCF_3_, Arachidonyl trifluoromethyl ketone), to clarify the potential role of different types of PLA2 isoform during SGIV infection [31,32]. After the evaluation of cell viability upon the treatment with these two inhibitors (Figure 4A), we found that PACOCF_3_ and AACOCF_3_ both showed significant impacts on SGIV replication, evidenced by the decrease in viral gene transcription (Figure 4D,E), protein synthesis (Figure 4C) and viral production. Specifically, the viral titer in PACOCF_3_- or AACOCF_3_-treated cell lysates was decreased up to 40% or 28.76% compared with those from DMSO- or ethanol-treated cells at 24 h p.i., respectively (Figure 4F,G). Thus, our results suggested that both cPLA2 and iPLA2 played critical roles in SGIV infection.

### 2.4. Grouper Cytosolic Phospholipase A2-Alpha (EccPLA2α) Was Essential for SGIV Infection

**Figure 5:**
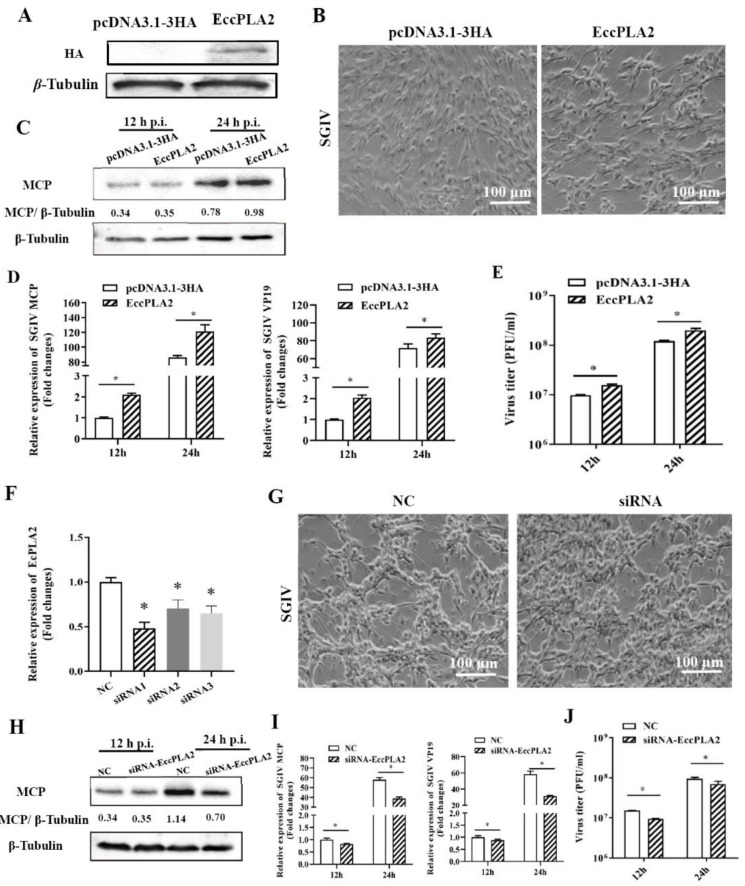
The effects of EcPLA2α overexpression or knockdown on SGIV replication. (**A**–**E**) Overexpression of EcPLA2α enhanced SGIV replication. (**F**–**J**) Knockdown of EcPLA2α suppressed SGIV replication. (**A**,**F**) EcPLA2α was successfully overexpressed or knocked down in transfected cells. The protein or transcription level of EcPLA2α in pcDNA3.1-3HA- or 3HA-EcPLA2α-transfected cells were detected by Western blot or qPCR. (**B**) The CPE induced by SGIV at 24 h p.i. was improved in EcPLA2α overexpressing cells. (**G**) The CPE induced by SGIV at 24 h p.i. was delayed in EcPLA2α knockdown cells. (**C**,**H**) Western blotting analysis of viral MCP during SGIV infection in EcPLA2α overexpressing or knockdown cells. (**D**,**I**) The effect of the overexpression or knockdown of EcPLA2α on SGIV MCP or VP19 transcription during SGIV was examined by qPCR. (**E**,**J**) The virus production of SGIV in infected cells was examined by virus plaque assay after EcPLA2α overexpression or knockdown in EAGA cells. Data are expressed as means ± SD. The asterisks (*) indicated *p* < 0.05.

As we observed the role of cPLA2 inhibitor in SGIV replication, we aimed to determine whether the critical molecule cPLA2α was involved in this process. First, we cloned the full-length cDNA of grouper cPLA2α (EccPLA2α) and constructed the recombinant plasmid (HA-EccPLA2α) for the ectopic expression of EccPLA2α in vitro. As shown in Figure 5A, EccPLA2α expression could be clearly examined in transfected GS cells. Next, the effects of EccPLA2α overexpression on SGIV infection were examined by Western blot, qPCR, and plaque assay. Compared to the control vector transfected cells, the CPE induced by SGIV was accelerated in EccPLA2α-overexpressing cells at 24 h p.i (Figure 5B). Consistently, qPCR results showed that the transcription levels of the SGIV, MCP, and VP19 genes were significantly increased in EccPLA2α-overexpressing cells at 24 h p.i (Figure 5D). Moreover, the overexpression of EccPLA2α significantly increased the protein synthesis of SGIV MCP and virus production (Figure 5E).

We also evaluated the roles of EccPLA2α in SGIV replication by knocked down EccPLA2α in vitro using specific siRNA. Compared with the negative control siRNA, siRNA1 showed the most significant silence efficiency of EccPLA2α at the transcription level and were chosen for the following experiments (Figure 5F). The results of qPCR and Western blot showed that the knockdown of EccPLA2α significantly inhibited SGIV infection (Figure 5G), evidenced by the marked decrease in transcription and expression of viral genes (Figure 5H,I). Consistently, knockdown of EccPLA2α also reduced the virus titer (Figure 5J). Together, it was speculated that EccPLA2α was essential for SGIV infection in vitro.

### 2.5. Inhibition of Arachidonic Acid Metabolism Impaired SGIV Replication

**Figure 6:**
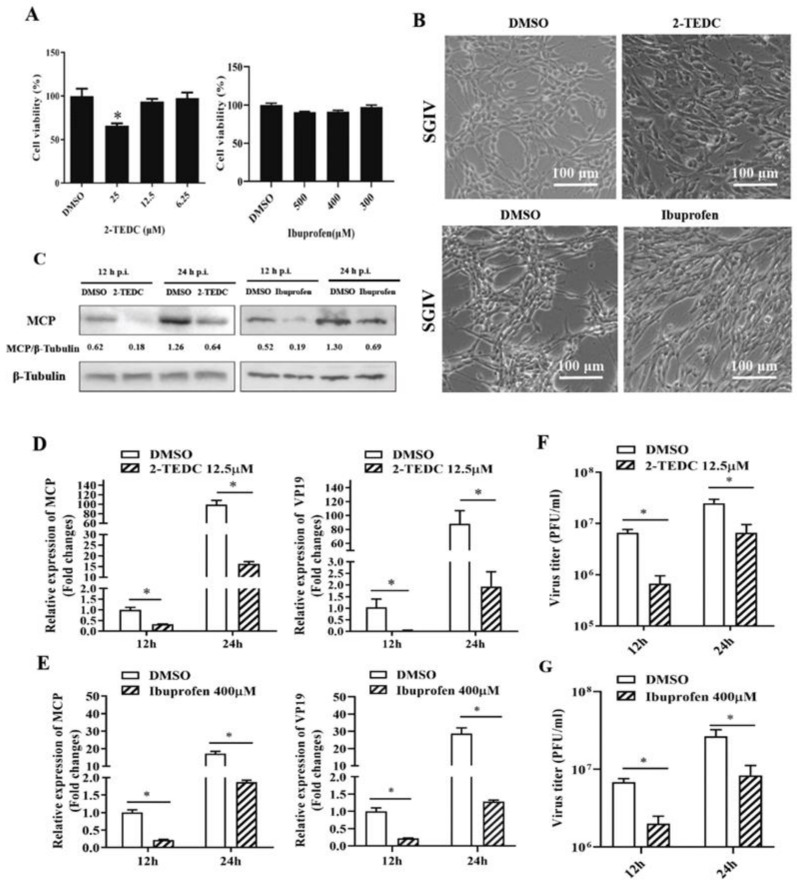
The roles of AA metabolism during SGIV replication. (**A**) The cytotoxicity of 2-TEDC or Ibuprofen on GS cells was determined using MTT assay. (**B**) The severity of CPE induced by SGIV infection at 24 h p.i. in 2-TEDC or Ibuprofen-treated cells. (**C**) Western blotting analysis of viral MCP during SGIV infection in 2-TEDC or Ibuprofen-treated cells. (**D**,**E**) The viral gene transcription levels of SGIV, MCP, and VP19 were detected in infected cells after treatment with 2-TEDC or Ibuprofen by qPCR, respectively. (**F**,**G**) The effects of 2-TEDC or Ibuprofen on virus titers during SGIV infection were detected by virus plaque assay. Data are expressed as means ± SD. The asterisks (*) indicated *p* < 0.05.

It was reported that PLA2 catalyzes the hydrolysis of membrane glycerophospholipids to liberate arachidonic acid (AA), and key classes of enzymes, including cyclooxygenases (COX) and lipoxygenases (LOX), can metabolize AA to produce biologically active eicosanoids [33]. To further verify the roles of AA metabolism in SGIV infection, 2-TEDC (a potent inhibitor of 5-, 12-, and 15-LOX) and Ibuprofen (an inhibitor of Cox-1 and Cox-2) were used in our study, and their effects on viral replication were determined. As shown in Figure 6, the CPE induced by SGIV was clearly delayed in the presence of 2-TEDC or Ibuprofen (Figure 6B). The transcription level of viral genes, including MCP and VP19, significantly decreased after treatment with 2-TEDC or Ibuprofen (Figure 6D,E). Furthermore, Western blot assay showed a marked reduction in MCP expression after treatment with 2-TEDC or Ibuprofen compared with DMSO (Figure 6C). The virus titers were also significantly reduced in 2-TEDC- or Ibuprofen-treated cells compared with DMSO-treated cells. Specifically, treatment with 2-TEDC- or Ibuprofen decreased the viral titers by up to 28.76% and 31.1% at 24 h, respectively (Figure 6F,G). Thus, these results showed that AA metabolism was also crucial for SGIV replication.

## 3. Discussion

Similar to other lager DNA viruses, iridovirus entry, uncoating, assembly, and egress were intimately associated with cell membrane system which composed of different lipid composition [3,6,8,25]. Many studies showed that cellular lipids played a vital role in multiple stages of the virus life cycle [9]. RNA viruses, such as dengue virus, West Nile virus, and coronavirus, alter lipid homeostasis and remodel the host cytoplasmic membrane system that results in the formation of membranous structures for virus assembly or viral genome replication [10,34,35]. As for SGIV, a large DNA virus, the host-cell lipid changes, in the context of virus infection, were proposed to be related to virus infection-induced apoptosis in GEC cells [26]. Interestingly, SGIV infection in several host cells evoked non-apoptotic cell death, and no apoptotic bodies were examined in infected cells [27,28]. Therefore, the lipidomic changes during SGIV infection, which induced non-apoptotic cell death, is worth investigating.

In the present study, the whole-cell lipidomic profiling during SGIV infection was analyzed using UPLC-Q-TOF-MS/MS. Our lipidomic data showed that the significant alterations in the lipid contents were detected upon SGIV infection. More than 40% of differential changed lipid species belonged to GPs. Given that GPs are major components of cellular membranes, we paid attention to the alteration of the lipid species involved in GP or AA metabolism. We found that GPs and FAs were significantly elevated in SGIV-infected cells, suggesting that SGIV infection disturbed GPs homeostasis. Moreover, the number of GPs species increased with the infection time. The majority of PS, PC, and PI species were markedly upregulated, and the half of PE and PA species were downregulated, while most PG species decreased in abundance in SGIV-infected cells. PS is highly enriched in the inner leaflet of the plasma membrane and in intracellular organelles such as endosomes, and acts as a tag for the recognition of apoptotic cells [36]. Our previous studies showed that SGIV infection induced cell death without the externalization of PS. We speculated that the increase in PS abundance might be linked to viral internalization and budding. PCs, Pes, and PSs were the primary components of most cellular membranes. These lipid species increased in abundance, especially at the peak of viral replication during dengue virus and red-spotted grouper nervous necrosis virus (RGNNV) infection [11,37]. On the other hand, tubular, membrane-like structures were observed in virus assembly sites in SGIV-infected cells [25]. These membrane-like structures derived from endoplasmic reticulum (ER) might be viral assembly intermediates. Thus, a hypothesis was proposed that SGIV might induce GPs remodeling, such as RNA viruses to form membrane-like structures that were required for virus replication and assembly. Interestingly, there were no significant changes in the abundance of lyso-GPs during SGIV infection. Differently, the majority of the phospholipid groups were down-regulated, with the exception of lyso-PChol during West Nile virus [17], while the level of lysoPC and lysoPE were up-regulated in human coronavirus 229E (HCoV-229E) [12]. The alterations of the GP contents might be related to its different actions in the response to the infection of different viruses.

Glycerophospholipid acyl chains were remodeled by reactions of PLAs and LPLATs to generate lysophospholipids and FAs [29]. Although there were no differential changes in the abundance of lysophospholipids, FAs were significantly elevated in SGIV-infected cells. Eicosanoids, as an inflammatory mediator, have multiple effects on inflammation and immunity [38]. In the present study, three prostaglandins (PGH2-EA, 15-keto-PGF2α, and PGF1β), one leukotriene (LTB4 ethanol amide) showed increased levels during SGIV infection. Our previous metabolomic studies showed that the level of oleic acid and linoleic acid also increased in SGIV-infected cells, which was consistent with our lipidomic data (data not published). Moreover, the increase in eicosanoids during SGIV infection might be related to the immune or inflammatory response induced by SGIV.

Given the major membrane rearrangements occurring in virus-infected cells, key enzymes involved in cellular GP metabolism were reported to play a vital role in this process. The investigations of GP remodeling mainly focused on PLAs [39]. Cytosolic PLA2α (cPLA2α) was identified as being involved in multiple cellular processes, especially in inflammatory response, and recently cPLA2 activity was reported as essential for virus infection. The specific inhibition of cPLA2 activity has detrimental effects on human coronavirus 229E replication [19]. Our results showed that the treatment with cPLA2-specific inhibitors showed an inhibitory effect on SGIV infection. Moreover, the results of grouper cPLA2α overexpression and knockdown further confirmed its action in SGIV infection. The similar roles of cPLA2α were also reported in the process of HCV and West Nile virus infection. The knockdown of PLA2G4C (named as cPLA2α) significantly suppressed HCV or WNV_KUN_ replication [17,20]. Nevertheless, the mechanism of action of cPLA2α in different viruses was diverse. cPLA2α was involved in the early infection of HCov-229E, and then affected the formation of replication organelles (ROs) [19]. The detailed mechanism of how cPLA2α affects SGIV replication needs to be further investigated.

Phospholipid hydrolysis is then metabolized by PLA2 generate AA through the COX and LOX pathways to produce prostaglandin (PGs), prostacyclins, thromboxanes, and leukotrienes [40]. Our lipidomic analysis showed that SGIV infection led to an increase in the abundance of prostaglandin and leukotrienes, including PGH2-EA, 15-keto-PGF2α, PGF1*β* and LTB4 ethanol amide. Prostaglandin can modulate the host defense against viruses. As two key enzymes involved in AA metabolism, COX and LOX also play essential roles in virus infection. The inhibition of COX activity in vesicular stomatitis virus (VSV) or pseudorabies virus (PRV)-infected cells led to a reduction in viral production [41,42]. The inhibitory effects on viral replication were also detected during SGIV infection after treatment with COX or 5-LOX inhibitors. During human cytomegalovirus (HCMV) infection, epidermal growth factor receptor (EGFR), c-Raf, mitogen-activated protein kinases (MEK1/2), and extracellular signal-regulated kinases (ERK1/2) pathways were involved in COX2-mediated inflammation induced by HCMV [43]. Our previous studies showed that SGIV infection activated MAPK signaling pathways, and ERK signaling participated in SGIV infection-induced nonapoptotic cell death and viral replication [27,44]. Whether the activation of MAPK signaling pathways is related to the action of COX during SGIV infection needs to be investigated in the further studies.

**Figure 7:**
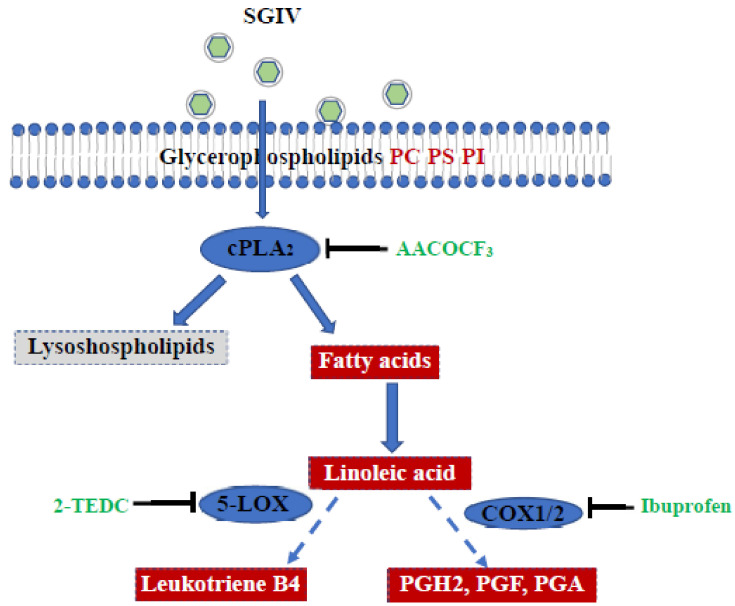
Model for the roles of cPLA2 and AA metabolism in SGIV infection. SGIV infection resulted in alteration of GPs homeostasis in vitro. Inhibition of cPLA2, COX, and 5-LOX significantly decreased SGIV replication. Inhibitors were shown in green. The key enzyme involved in GPs and AA metabolism were shown in blue. The lipid metabolites, which increased during SGIV infection, were shown in red. The lipid metabolites, which were not significantly changed in SGIV-infected cells, were shown in gray.

The present study investigated whole-cell lipidomic profiling during SGIV infection using UPLC-Q-TOF-MS/MS. The lipidomic results showed that glycerophospholipids (GPs) and fatty acids (FAs) were significantly elevated in SGIV-infected cells, suggesting that SGIV infection disturbed GPs homeostasis, and then affected the metabolism of FAs, especially arachidonic acid (AA). Furthermore, the inhibition of crucial enzymes involved in GPs or AA metabolism, including cPLA_2_, 5-LOX, or COX, all significantly suppressed SGIV replication, indicating cPLA_2_ activity and AA metabolism were essential for SGIV infection (Figure 7). The role of cPLA_2_ activity in SGIV infection was further confirmed when grouper cPLA2α (EccPLA2α) was overexpressed or knocked down in vitro. Our results not only extend the current understanding of interaction between SGIV and host-cell GP and AA metabolism, but also shed important light on SGIV pathogenesis.

## 4. Materials and Methods

### 4.1. Cell Culture, Virus, and Reagents

The grouper spleen (GS) cells were established and maintained in our lab [45]. GS cells were grown in Leibovitz’s L15 medium containing 10% fetal bovine serum (Gibco) at 28 ℃. The SGIV used in the study was prepared in GS cells as described previously [44]. For SGIV infection, the cells were infected with the virus at a multiplicity of infection (MOI) of 1 in the following experiment.

Anthranilic acid (ACA) was purchased from APExBIO. YM26734, PACOCF3, and TEDC-2 was purchased from Tocris Bioscience, and Ibuprofen was purchased from MedChemExpress (MCE). The above reagents were dissolved in DMSO. AACOCF3 was purchased from Good Laboratory Practice bioscience (GLPBIO) and was dissolved in ethanol. The cytotoxicity of reagents was determined in GS cells using 3-(4,5-dimethylthiazol-2-yl)-2,5-diphenyltetrazolium bromide (MTT) assay.

### 4.2. Virus Infection

GS cells were grown in 24-well plates or 6-well plates and pretreated with DMSO, ethanol or proper concentrations of different reagents for 2 h. Then, the cells were infected with SGIV at 1 MOI and cultured at 28 ℃. At indicated infection time, the cytopathic effect (CPE) of cells was observed under the microscopy (Zeiss). At the indicated time, the expression of viral major coat protein (MCP) was examined by Western blotting, and the transcription levels of viral MCP and VP19 were determined by quantitative PCR (qPCR). The whole-cell lysates of infected cells were collected at indicated time for virus titer assay. The experiments were independently carried out three times.

For analysis of lipidomic analysis, 1 × 10^7^ GS cells were grown to confluence for 18 h. Cells were then washed with serum-free L15 medium before infection or mock treatment. GS cells were infected with SGIV at 1 MOI. GS cells were incubated with the equivalent medium as a mock infection. Mock- and SGIV-infected cells (n = 8) were harvested for lipidomic analysis at 24 h and 48 h post infection (p.i.), respectively. The samples were centrifuged at 1200 rpm/min for 10 min, and cell pellets were frozen in liquid nitrogen until used.

### 4.3. Lipidomic Analysis

Lipids were extracted from an equal number of cells as follows: (1) The cell pellets were resuspended in 600 μL methanol–water, and then transferred into 10 mL vial, then 20 μL of internal standard (Lyso PC17: 0, 0.01 mg/mL, methanol configuration) were added; (2) Sonicate was performed in ice bath for 10min at 500 W; (3) The homogenate was transferred to LC-MS injection vial, and was stood at −4 °C for 30 min; (4) The liquid was stratified, and the lower liquid (Chloroform layer) was transferred into another LC-MS injection vial, and vacuum dry; (5) An amount of 600 μL of chloroform–methanol (*V:V* = 2:1) was added to the injection vial after taking the lower layer solution and vortexed for 30 s; ultrasonic extraction for 10 min at −20 °C; (6) The mixture was stood at −4 °C for 30 min; the lower liquid was taken and placed in the original LC-MS vial and vacuum dried; (7) After drying, the lipid residue in the LC-MS vial was reconstituted with 300 μL isopropanol–methanol (*V:V* = 1:1) (vortex for 30 s, ultrasonic extraction for 3 min), and transfer the solution to a 1.5 mL EP tube. The solution was centrifuged for 10 min (13,000 rpm, 4 °C), and 200 μL of supernatant was added to the LC-MS sample vial for LC-MS analysis; (8) Quality control samples (QC) were prepared by mixing equal volumes of extracts of all samples, and the volume of each QC was the same as the sample.

The samples were separated by Nexera UPLC (Shimadzu, Kyoto, Japan) system. Chromatographic conditions: column temperature: 45 °C; mobile phase A: acetonitrile: water (60:40, *V/V*), solution contains 10 mmol/L ammonium formate, 0.1% formic acid; mobile phase B: acetonitrile: isopropanol (10:90, *V/V*), the solution contains 10 mmol/L ammonium formate, 0.1% formic acid; flow rate: 0.35 mL/min; injection volume: 5 μL.

Mass spectrometry was performed by Q exactive mass spectrometer (Thermo Scientific™). The heating electrospray ionization (HESI) positive and negative ion mode was used to detect the lipid components. Mass spectrum conditions—Positive: Heater Temp 300 °C, Sheath Gas Flow rate 45 arb, Aux Gas Flow Rate15 arb, Sweep Gas Flow Rate 1arb, spray voltage 3.5 KV, Capillary Temp 320 °C, S-Lens RF Level 50%. MS1 scan ranges: 120–1800. Negative: Heater Temp 300 °C, Sheath Gas Flow rate 45 arb, Aux Gas Flow Rate 15arb, Sweep Gas Flow Rate 1arb, spray voltage 3.1 KV, Capillary Temp 320 °C, S-Lens RF Level 50%. MS1 scan ranges: 120–1800. The mass-to-charge ratios of lipid molecules and lipid fragments were collected as follows. Ten fragment maps (from the MS2 scan, HCD) were collected after each full scan. An MS1 resolution set to 70,000 at *m*/*z* 200, and an MS2 resolution set to 17,500 at *m*/*z* 200.

The acquired LC-MS raw data were analyzed by the progenesis QI v2.3 software (Nonlinear Dynamics, Newcastle, UK). The identification of compounds was based on the accurate mass number, secondary fragments, and isotopic distribution. HMDB and lipidmaps (v2.3) databases were used for qualitative analysis. The extracted data were normalized by total peak area, and then subjected to multivariate analysis (MVA) using SIMCA-P 14.1 software (Umetrics, Umea, Sweden). Principle component analysis (PCA) and (orthogonal) partial least-squares-discriminant analysis (OPLS-DA) were performed to test the lipid molecules alterations between the mock group and virus groups. The differentially expressed metabolites were selected according to two criteria: *p*-value (<0.05) and fold change (FC) > 2 or FC < 0.5.

### 4.4. Cell Transfection

To evaluate the roles of EccPLA2 in SGIV infection in vitro, the full length of EccPLA2 (aa1-749) was cloned into pcDNA3.1-3HA using the primers in Table 1. The recombinant plasmid (HA-EccPLA2) was subsequently sequenced by DNA sequencing. GS cells were transfected with pcDNA3.1-3HA or 3HA-EccPLA2 for 24 h using Lipofectamine 2000 reagent (Invitrogen) according to the manufacture’s protocol. The cells were infected with SGIV at 1 MOI. The infected cells were harvested at 12 h or 24 h p.i. for RNA extraction, qPCR and Western blotting. In addition, knockdown of EccPLA2 was performed using specific, small, interfering RNA (siRNA) oligonucleotides. In brief, three siRNA targeting different sequences of EccPLA2 were designed and commercially synthesized by Suzhou GenePharma (Table 1). GS cells were transfected with siRNA-EccPLA2 or negative control (NC) at 160 nM for 24 h using Lipofectamine™ RNAiMAX Transfection Reagent (Invitrogen) according to the manufacture’s protocol. The transfected cells were infected with SGIV (MOI = 1) for further 12 h or 24 h. The infected cells were harvested for RNA extraction, qPCR and Western blotting.

### 4.5. Virus Titer Assay

The virus titers in whole-cell lysates of infected cells were determined on monolayers of GS cells by an agar overlay plaque assay. GS cells were pre-treated with DMSO, ethanol or different inhibitors (50 μM ACA, 25 μM AACOCF3, 12.5 μM PACOCF3, 20 μM YM26734, 12.5 μM 2-TEDC, 400 μM Ibuprofen) for 2 h, and then infected with SGIV at an MOI of 1. At 12 h or 24 h p.i. The whole-cell lysates of infected cells were collected to determine the cell associated virus production as described previously [27]. In brief, GS cells were grown in 24 wells for 18 h. The cell lysates were serially diluted by 10-fold and overlaid on monolayers of GS cells. After incubation for 1 h, the diluent was discarded, and 700 μL 0.75% agar was added into each well. Cells were infected for further 6 days at 28 ℃. Viral concentration was calculated through counting the plaques produced by the virus, and virus titer was represented as PFU/mL.

### 4.6. Western Blotting

At the indicated time points, SGIV-infected cells were harvested and the pellets were resuspended in 1× Pierce IP Lysis Buffer (Pierce). SDS-PAGE and Western blotting were performed as described previously [27]. Equal amounts of protein were subjected to SDS-PAGE and then transferred to polyvinylidene difluoride (PVDF) membranes. After blocking with 5% skimmed milk, the membranes were incubated with anti-*β*-tubulin, anti-HA, or anti-SGIV MCP at a dilution of 1:2000 for 2 h at room temperature. After washing with TBST, the membranes were incubated for 2 h with the HRP-goat, anti-mouse IgG or HRP-goat, anti-rabbit IgG (1:5000) at room temperature. Immunoreactive bands were visualized using an enhanced HRP-DAB chromogenic substrate kit (Tiangen, China), and the protein intensities were quantified using ImageJ software.

### 4.7. RNA Extraction, cDNA Synthesis, and Quantitative PCR (qPCR)

To determine the effects on different inhibitors on virus replication, viral gene transcriptions were detected by qRT-PCR. Total RNA from SGIV-infected cells were isolated using the cell total RNA isolation kit (Foregene) according to manufacturer’s instructions. The RNA was reverse transcribed using ReverTra Ace RT Kit (Toyobo). Amplification was examined using SYBR Green I Reaction Mix (Toyobo) in an Applied Biosystems QuantStudio 5 Real Time Detection System (Thermo Fisher). Each assay was carried out under the following cycling conditions: 95 °C for 5 min for activation, followed by 40 cycles at 95 °C for 5 s, 60 °C for 10 s, and 72 °C for 15 s. The primers used were listed in Table 1. The expression levels of target genes were standardized to β-actin and calculated with the 2^−△△^CT method. The data were indicated as mean ± SD and shown from one representative experiment carried out in triplicate. Statistical significance was determined with Student’s *t*-test and the statistical significance was set at *p* < 0.05.

## Figures and Tables

**Table 1:** Primers used in this study.

Primer Names	Sequence (5′-3′)
EcPLA2α-3HA-KpnI-F	CGGGGTACCATGGCTTCCAATATAATTGTGGA
EcPLA2α-3HA-BamHI-R	CGCGGATCCCTGGTTATCAGTTCCCAGGA
siRNA1-EcPLA2α	GCAGCAGUUCUCUCACAAATT
siRNA2-EcPLA2α	GCACAACAUCCUGGAGUUATT
siRNA3-EcPLA2α	GGUGGAGUUCAGCCCGUAUTT
EcPLA2α-RT-F	CAGTGATGGTGGTTCG
EcPLA2α-RT-R	ATGTTGTGCTGGTTGG
Actin-RT-F	TACGAGCTGCCTGACGGACA
Actin-RT-R	GGCTGTGATCTCCTTCTGCA
SGIV MCP-RT-F	GCA CGCTTCTCTCACCTTCA
SGIV MCP-RT-R	AACGGCAACGGGAGCACTA
SGIV VP19-RT-F	TCCAAGGGAGAAACTGTAAG
SGIV VP19-RT-R	GGGGTAAGCGTGAAGAC

## Data Availability

Data supporting the reported results can be provided by X.H. Huang.
